# A repeated cross-sectional study of clinicians’ use of psychotherapy techniques during 5 years of a system-wide effort to implement evidence-based practices in Philadelphia

**DOI:** 10.1186/s13012-019-0912-4

**Published:** 2019-06-21

**Authors:** Rinad S. Beidas, Nathaniel J. Williams, Emily M. Becker-Haimes, Gregory A. Aarons, Frances K. Barg, Arthur C. Evans, Kamilah Jackson, David Jones, Trevor Hadley, Kimberly Hoagwood, Steven C. Marcus, Geoffrey Neimark, Ronnie M. Rubin, Sonja K. Schoenwald, Danielle R. Adams, Lucia M. Walsh, Kelly Zentgraf, David S. Mandell

**Affiliations:** 1 Department of Psychiatry, Perelman School of Medicine, University of Pennsylvania, Philadelphia, PA USA; 2 Department of Medical Ethics and Health Policy, Perelman School of Medicine, University of Pennsylvania, Philadelphia, PA USA; 3 Penn Implementation Science Center at the Leonard Davis Institute of Health Economics (PISCE@LDI), University of Pennsylvania, Hall- Mercer Community Mental Health Center, Philadelphia, PA USA; 4 School of Social Work, Boise State University, Boise, ID USA; 5 Department of Psychiatry, University of California San Diego, San Diego, CA USA; 6 Department of Family Medicine and Community Health, University of Pennsylvania Perelman School of Medicine, Philadelphia, PA USA; 7 American Psychological Association, Washington, DC, USA; 8 Community Behavioral Health, Impact Reach, LLC, Philadelphia, PA USA; 9 Department of Behavioral Health, Philadelphia, PA USA; 10 Department of Child and Adolescent Psychiatry, New York University Langone Health, New York, NY USA; 11 School of Social Policy and Practice, University of Pennsylvania, Philadelphia, PA USA; 12 Oregon Social Learning Center, Eugene, OR USA; 13 School of Social Service Administration, University of Chicago, Chicago, IL USA; 14 Department of Psychology, University of Miami, Miami, FL USA; 15 Hall- Mercer Community Mental Health Center, Philadelphia, FL USA; 16 Impact Reach, LLC, Philadelphia, PA USA

**Keywords:** System-level implementation, Organizational factors, Cognitive-behavioral therapy, Targets

## Abstract

**Background:**

Little work investigates the effect of behavioral health system efforts to increase use of evidence-based practices or how organizational characteristics moderate the effect of these efforts. The objective of this study was to investigate clinician practice change in a system encouraging implementation of evidence-based practices over 5 years and how organizational characteristics moderate this effect. We hypothesized that evidence-based techniques would increase over time, whereas use of non-evidence-based techniques would remain static.

**Method:**

Using a repeated cross-sectional design, data were collected three times from 2013 to 2017 in Philadelphia’s public behavioral health system. Clinicians from 20 behavioral health outpatient clinics serving youth were surveyed three times over 5 years (*n* = 340; overall response rate = 60%). All organizations and clinicians were exposed to system-level support provided by the Evidence-based Practice Innovation Center from 2013 to 2017. Additionally, approximately half of the clinicians participated in city-funded evidence-based practice training initiatives. The main outcome included clinician self-reported use of cognitive-behavioral and psychodynamic techniques measured by the Therapy Procedures Checklist-Family Revised.

**Results:**

Clinicians were 80% female and averaged 37.52 years of age (*SD* = 11.40); there were no significant differences in clinician characteristics across waves (all *p*s > .05). Controlling for organizational and clinician covariates, average use of CBT techniques increased by 6% from wave 1 (*M* = 3.18) to wave 3 (*M* = 3.37, *p* = .021, *d* = .29), compared to no change in psychodynamic techniques (*p* = .570). Each evidence-based practice training initiative in which clinicians participated predicted a 3% increase in CBT use (*p* = .019) but no change in psychodynamic technique use (*p* = .709). In organizations with more proficient cultures at baseline, clinicians exhibited greater increases in CBT use compared to organizations with less proficient cultures (8% increase vs. 2% decrease, *p* = .048).

**Conclusions:**

System implementation of evidence-based practices is associated with modest changes in clinician practice; these effects are moderated by organizational characteristics. Findings identify preliminary targets to improve implementation.

## Background

The last two decades have shown increasing emphasis on the implementation of evidence-based practices (EBPs) in publicly funded behavioral health systems nationally [1, 2]. Policy-makers in public behavioral health systems (e.g., City of Philadelphia, Los Angeles County, Washington, Hawaii, New York) have committed to using EBPs [1, 3–6] to improve the quality of psychosocial services and client outcomes [7–9] using various approaches including tying reimbursement to EBP use (i.e., financial incentives), building EBPs into contracts, and policy initiatives [2, 10, 11]. Although many public behavioral health systems have invested in implementing EBPs, very few of these efforts have been systematically and rigorously evaluated, thus limiting the ability to understand the effect of these efforts on clinician and organizational behavior and subsequent client reach [2]. Thought leaders in implementation science have recommended learning from natural experiments enacted by systems via observational research designs in order to produce generalizable knowledge to advance implementation science [12]. Thus, rigorous evaluation of system-wide EBP implementation can produce valuable information to achieve this objective.

The majority of what is known about system-wide EBP implementation is largely descriptive in nature (i.e., if systems are implementing EBPs, how they support EBPs). One set of studies takes a broad perspective and reports on national trends across system EBP implementations. For example, a study reporting on a set of national surveys conducted with state mental health directors found increases in states offering EBPs for youth from 2001 to 2012 [2]. Another survey study found that the majority of state mental health directors endorsed using financial incentives to promote EBP use in their system [11]. Another set of studies takes a more granular perspective and reports on specific strategies used within one system such as the City of Philadelphia [10], New York [11], Hawaii, and Illinois [13]. For example, Powell and colleagues describe how the City of Philadelphia Department of Behavioral Health and Intellectual Disability Services (DBHIDS), which oversees behavioral health services for over 600,000 Medicaid-enrolled consumers, began implementing EBPs in 2007 via “EBP initiatives” [10, 14] and through the creation of the Evidence-based Practice and Innovation Center (EPIC) which included policy, fiscal, and operational changes to encourage EBP implementation [10].

Although these perspectives have enriched the field’s understanding of whether systems are implementing EBPs and how EBPs are supported, there is a gap in the literature with regard to how system-wide efforts to implement EBPs are related to clinician practice over time. Only a few studies have attempted to evaluate the effect of system-wide EBP implementation. One national study within the Veterans Health Administration, a large system supporting EBP implementation, found that medical record documentation suggested that only 20% of veterans with post-traumatic stress disorder (PTSD) (total *n* = 255,968) received at least one session of EBP for PTSD. Another study using administrative claims data found that there was an increased rate of EBP claims over time within the context of a fiscally mandated implementation effort in Los Angeles County [15]. By leveraging existing data sources, these studies provide preliminary insights into how system-wide efforts to implement EBPs may be related to patterns in clinician and organizational behavior, but additional work is needed to understand the effect of such efforts.

Another focus of research inquiry includes investigating how system implementation of EBPs interacts with characteristics of the organizations nested within the system, such as organizational leadership, culture, and climate. This line of research can both elucidate potential mutable targets of implementation strategies in future implementation efforts and advance the science of implementation by providing empirical evidence for implementation science frameworks that posit the criticality of these constructs [16]. Leading determinant frameworks [17] such as the Consolidated Framework for Implementation Research [18] and the Exploration, Preparation, Implementation, and Sustainment framework [16] suggest the importance of the relationship between implementation and organizational characteristics, such as leadership (i.e., extent to which leaders are capable of guiding, directing, and supporting implementation) [19], culture (i.e., shared norms, behavioral expectations, and values of an organization) [20, 21], and climate (i.e., shared perceptions regarding the impact of the work environment on clinician well-being). A growing body of literature explores the relationship between these constructs and implementation (e.g., [19, 22–27]). However, findings have been somewhat mixed [19] and few studies have prospectively investigated the relationship between these factors and implementation—which would provide the most compelling evidence for potential mutable targets of implementation strategies, as well as build causal theory, a key imperative in implementation science [19, 28, 29].

The current study builds on previous work by investigating how a centralized system effort to support implementation in the City of Philadelphia is related to clinicians’ EBP use and how organizational characteristics, specifically implementation leadership, implementation climate, and organizational culture, might moderate these effects [16, 23, 30]. We measured clinicians’ self-reported use of psychotherapy techniques for youth in outpatient clinics over 5 years within the context of a system-wide effort to implement EBPs. We measured cognitive-behavioral therapy (CBT) techniques, which have evidence for their effectiveness for youth psychiatric disorders [31] and comprised the majority of EBPs implemented by DBHIDS, and psychodynamic techniques, which are frequently used [32] but have less evidence for youth psychiatric disorders [31, 33–37]. We hypothesized that (a) clinician CBT use would increase over time, whereas psychodynamic technique use would remain static; (b) clinician participation in system-sponsored EBP initiatives would increase CBT use over time; and (c) baseline organizational variables would predict variability in clinician CBT use over time [14].

## Methods

### Design

We used a repeated cross-sectional design [38] across 5 years of system-wide EBP implementation in which we were interested in change in technique use at the population level and the moderating effect of organization-level variables on those estimates. In a repeated cross-sectional design, there may be zero overlap in the samples between periods and yet valid inferences of change in population values can be made on the basis of repeated cross-sections. Additionally, the effect of organizational moderators can be examined as long as the same organizations are in the sample. Overlap in the cross-sectional samples is beneficial because it reduces variance of the parameter estimates; however, high overlap between samples is not necessary for valid inferences about changes in population trends over time.

The design incorporated two sampling stages. First, we purposively selected organizations delivering youth outpatient services in Philadelphia’s public behavioral health system. Second, cross-sections of clinicians working within sampled organizations at each wave were recruited. At each wave (2013, 2015, 2017), we attempted to recruit all clinicians within enrolled organizations. This allowed us to examine changes in the population of interest over time without assuming that individuals were the same at each wave, given high clinician turnover rates and the real-world context.

### Procedure

With the permission of organizational leaders, researchers scheduled group meetings with all clinicians working within the organizations that delivered youth outpatient services, during which the research team presented the study, obtained written informed consent, and collected measures onsite. The only inclusion criterion was that clinicians deliver behavioral health services to youth (clients under age 18) via the outpatient program. We did not exclude any clinicians meeting this criterion and included calinicians-in-training (e.g., interns); the majority of clinicians had their master’s degree. Clinicians received $50 each wave; clinicians participating in all three waves received an additional $50. Procedures were approved by the University of Pennsylvania and City of Philadelphia IRBs.

### Setting

**Fig. 1:**
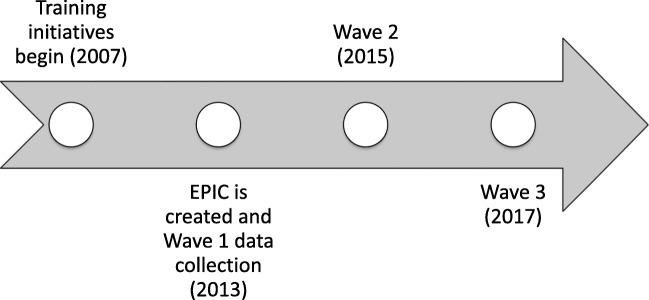
Timeline of evidence-based practice initiatives, Evidence-Based Practice Innovation Center (EPIC), and data collection

Prior to 2013, DBHIDS supported EBPs via separate “EBP initiatives” that included training and expert consultation for enrolled clinicians lasting approximately 1 year, as recommended by treatment developers [10]. Between 2007 and 2019, through these initiatives, DBHIDS supported the implementation of a variety of cognitive behavioral therapy-focused practices addressing a range of psychiatric disorders including cognitive therapy [39], prolonged exposure [40], trauma-focused CBT [10, 41], dialectical behavior therapy [42], and parent-child interaction therapy [43] (all currently ongoing; see Fig. 1). Initially, DBHIDS largely guided organization selection for initiative participation; more recently, organizations have applied for participation through a competitive process. Organizations decided which of their clinicians would participate [44].

As system leaders identified similar barriers across single EBP initiatives, the DBHIDS Commissioner (ACE) convened a task force of academics and policy-makers in 2012 to apply best practices from implementation science to support EBP implementation. This resulted in EPIC, an entity intended to provide a centralized infrastructure for EBP administration. EPIC was formally launched in 2013 and oversees all EBP implementation efforts in the Philadelphia public behavioral health system. EPIC is led by a Director and currently supported by two staff who provide technical assistance to organizations around EBP implementation through meetings, telephone calls, and regular events. In addition to supporting the EBP initiatives, which predated the creation of this centralized infrastructure, EPIC aligned policy, fiscal, and operational approaches by developing systematic processes to contract for EBP delivery, hosting events to publicize EBP delivery, designating providers as EBP agencies, and creating enhanced rates for the delivery of some EBPs. For more details on the approach taken by EPIC and DBHIDS, please see [10]. All organizations and clinicians were exposed to system-level support provided by EPIC from 2013 to 2017. Data collection in 2013 occurred prior to the official launch of EPIC.

### Participants

#### Organizations

We used a purposive sampling approach for organizational recruitment. Philadelphia has a single payer system (Community Behavioral Health; CBH) for public behavioral health services, thus we obtained a list from the payer of all organizations that had submitted a claim in 2011–2012. There were over 100 organizations delivering outpatient services to youth. Our intention was to use purposive sampling to generate a representative sample of the organizations that served the largest number of youth in the system. We selected the first 29 organizations as our population of interest because together they serve approximately 80% of youth receiving publically funded behavioral health care. Over the course of the 5 years, we enrolled 21 out of the 29 organizations (73%; some organizations had more than one site, resulting in a total of 27 sites). Sixteen organizations and 20 sites were enrolled in the study at baseline and participated in at least one additional wave and were included in our analysis (*k* = 20). The organizations included in this study served approximately 42% of youth receiving outpatient services through the public mental health system (total) and represented approximately 52% of youth receiving outpatient services in the purposive sample of organizations that we targeted for recruitment. Organizations were geographically spread across Philadelphia and ranged in size with regard to youth served annually (*M* = 772.27, range = 337–2275 youth).

#### Clinicians

The three cross-sectional samples included 112 clinicians at wave 1 (46% organizational response rate), 164 clinicians at wave 2 (65% organizational response rate), and 151 clinicians at wave 3 (69% organizational response rate; total *N* = 340). Each cross-sectional sample included new clinicians and previous participating clinicians; 259 clinicians (76%) provided data once, 65 clinicians (19%) provided data twice, and 16 clinicians (5%) provided data three times [45, 46].

### Measures

#### Dependent variable

Use of CBT and psychodynamic techniques was measured using the Therapy Procedures Checklist-Family Revised (TPC-FR) [47], a 62-item self-reported checklist of clinician practice. At each wave, clinicians reported on the specific psychotherapy techniques they used with a current, representative client. All items are rated from 1 (*rarely*) to 5 (*most of the time*). The factor structure has been confirmed, and the instrument is sensitive to within-therapist changes in strategy use [47, 48]. Only the CBT (33 items; α = .93) [49] and psychodynamic subscales (16 items; α = .85) were used.

#### Independent variable

Formal participation in system-sponsored EBP initiatives was reported by clinicians in response to a series of questions asking if they “formally participated as a trainee through DBHIDS” in the five EBP initiatives (yes/no). To ensure accuracy, we confirmed with each participant that they understood that the questions referred to the 1-year training and ongoing consultation provided by DBHIDS for each initiative. In analyses, this variable was included as a time-varying continuous value indexing the cumulative number of initiatives the clinician had participated in up to each wave (range = 0 to 5). As a control variable, we also created a dichotomous variable indexing whether the clinician had participated in a system-sponsored EBP initiative prior to study entry (yes/no).

#### Organizational moderators of interest

Organizational measures were constructed by aggregating (i.e., averaging) individual responses within the organization after confirming high levels of within-organization agreement using average within-group correlation (*a*_*wg*_, *r*_*wg*_) statistics [50]. These statistics indicate the extent to which clinicians within an organization exhibit absolute agreement with each other on their ratings of organizational characteristics. Values range from 0 to 1, where higher values indicate greater reduction in error variance, and hence higher level of agreement. Typically, a cutoff of .6 or higher is recommended to provide validity evidence for aggregating individual scores to the organization level [51, 52].

Proficient organizational culture was measured using the 15-item *proficiency* scale (α = .92) of the Organizational Social Context measure [53]. Proficient organizational culture has been theoretically linked to EBP implementation [54]; items refer to shared norms and expectations that clinicians place client well-being first, are competent, and have up-to-date knowledge. Proficiency scale scores demonstrate excellent reliability, criterion-related validity, and predictive validity [23, 53–56]. Items are scored on a 1 (*never*) to 5 (*always*) scale.

Implementation leadership was measured using the Implementation Leadership Scale (ILS) [57], a 12-item scale that measures leader proactiveness (α = .92), knowledge (α = .97), supportiveness (α = .96), and perseverance (α = .95) in EBP implementation. Given high inter-correlations across ILS subscales and for parsimony, we used the total score (α = .98) only, which is supported by psychometric work [57]. Psychometrics suggest excellent internal consistency and convergent and discriminant validity [57]. Items are scored on a 0 (*not at all*) to 4 (*very great extent*) scale.

Implementation climate was measured using the Implementation Climate Scale (ICS) [58], an 18-item measure of strategic climate around EBP implementation. The six subscales on the ICS measure include organizational focus on EBP (α = .91); educational support for EBP (α = .86); recognition for using EBP (α = .86); rewards for using EBP (α = .87); selection of staff for EBP (α = .93); and selection of staff for openness (α = .95) [59]. The ICS produces subscale scores for each of these factors in addition to a total score. Given high inter-correlations across ICS subscales and for parsimony, we used the total score (α = .94) only, which is supported by psychometric work [58]. Psychometric data suggest good reliability and validity [58]. Items are scored on a 0 (*not at all*) to 4 (*very great extent*) scale.

#### Covariates

Covariates were included in the models on the basis of theory and prior research showing that client age [45], organizational size [60], clinician demographic characteristics (i.e., age, gender, educational level, years of clinical experience, experience in an EBP initiative prior to baseline [23]), and clinician attitudes toward EBPs (as measured by the Evidence-Based Practice Attitude Scale (EBPAS) [61]) are related to clinicians’ use of psychotherapy techniques [23, 62–65].

### Data analysis plan

We used three-level mixed effects regression models with a Gaussian distribution to generate estimates of clinicians’ average technique use at baseline and over time [66]. Models were estimated via full information maximum likelihood [67] in HLM 6.08 [68] and incorporated random intercepts and a random effect for time at the clinician and organization levels to account for the nested data structure. Preliminary analyses indicated that the optimal functional form for time was a single linear trend based on model comparisons using Schwarz’s Bayesian information criterion (BIC) where lower values indicate superior fit. Differences in BIC values of 5.6 (for CBT use) and 3.4 (for psychodynamic technique use) provided positive evidence for the superiority of the linear trend model relative to quadratic and categorical parameterizations of time [69]. Table 1 presents the raw means of each dependent variable by wave. Preliminary analyses also confirmed there was significant variance in clinicians’ use of CBT techniques (ICC (1) = .17, *p* < .001) and psychodynamic techniques (ICC (1) = .09, *p* < .001) at the organization level. All analyses controlled for all covariates described above (i.e., client age, clinician age, gender, education, years of experience, attitudes toward EBP, participation in EBP initiative prior to study entry, and organization size) [23, 44, 45]. No data were missing at the organization level. Clinician-level covariates had < 4% missingness; results of Little’s MCAR test indicated that they were missing completely at random (χ^2^ = 26.10, *df* = 22, *p* = .247); we imputed these covariate values using the serial mean [66].

**Table 1:** Descriptive statistics for dependent and independent variables

	Wave 1 (*n* = 112)		Wave 2 (*n* = 164)		Wave 3 (*n* = 151)	
	Mean (or %)	*SD*	Mean (or %)	*SD*	Mean (or %)	*SD*
Dependent variables						
Clinician level						
Use of CBT techniques (1–5)	3.25	.70	3.33	.67	3.40	.61
Use of psychodynamic techniques (1–5)	3.41	.66	3.38	.67	3.49	.65
Independent variables						
Clinician level						
Cumulative # of EBP initiatives clinician participated in (0–5)	.63	.86	.74	.99	1.12	1.27
Years of clinical experience	8.80	7.43	8.99	7.43	7.89	7.04
Clinician age in years	38.41	11.91	38.54	12.04	37.18	11.06
Clinician attitudes toward EBP (0–4)	2.93	.51	2.94	.48	2.90	.49
Client age in years	10.70	3.68	10.70	3.48	11.05	3.26
Participated in EBP initiative prior to study entry? (yes/no)	42%		38%		50%	
Education level						
Bachelor’s degree	5%		7%		7%	
Master’s degree	86%		83%		83%	
Doctoral degree	10%		10%		11%	
Female gender	75%		77%		83%	
Organization level (*N* = 20)						
Organization size (# child clients served per year)^a^	678	490				
Proficient culture (T-score, μ = 50, σ = 10)	48.63	13.08				
Implementation climate—recognition (0–4)	1.91	.84				
Implementation climate—reward (0–4)	.54	.51				
Implementation leadership—proactive (0–4)	2.33	.74				
Implementation leadership—knowledgeable (0–4)	2.84	.65				
Implementation leadership—supportive (0–4)	3.04	.73				
Implementation leadership—perseverant (0–4)	2.81	.75				

^a^Variable was divided by 100 for analysis

Average change in clinicians’ self-reported use of psychotherapy techniques (hypothesis 1) was tested in models with a linear main effect for wave and covariates. This estimated the overall change in clinicians’ use of psychotherapy techniques across waves. The influence of clinician participation in EBP initiatives on use of psychotherapy techniques (hypothesis 2) was tested by adding a time-varying variable which indexed the cumulative number of initiatives each clinician had participated in at each wave (0 to 5). Relationships between organizational variables of interest at baseline and subsequent trends in clinicians’ average use of therapy techniques over time (hypothesis 3) were tested by adding a main effect and interaction term for each organizational moderator. Because of high inter-correlations among organizational characteristics (mean *r* = .66), each organizational characteristic was tested separately. The issue of multiple comparisons is complex and contested [70, 71]. To avoid type II errors and the premature closing of important lines of inquiry, we used the Benjamini-Hochberg (B-H) procedure with a false discovery rate of .25 to evaluate the statistical significance of moderator tests for each outcome variable [72]. For each moderator test, we report the results of the B-H test of statistical significance, unadjusted and adjusted *p* values, and measures of effect size. Effect sizes were calculated using two metrics. First, we calculated percent change in technique use from wave 1 to wave 3. Second, we calculated a standardized mean difference in technique use from wave 1 to wave 3. After specifying each model, we examined residuals at levels 1, 2, and 3 and confirmed the tenability of underlying statistical assumptions including normality, homoscedasticity, and functional form [67].

## Results

### Clinician demographics

Clinicians were 80% female (*n =* 271), averaged 37.52 years of age (*SD* = 11.40), and had 8.37 (*SD* = 7.19) years of experience. Sixteen percent of clinicians endorsed identifying as Hispanic/Latino. With regard to race, clinicians endorsed identifying as White (43.5%), Black (30.6%), Asian-American (5.9%), American Indian or Alaska Native (1.2%), and Other (16.5%). Racial information was missing or not reported for 2.4% of the sample. Most clinicians had a master’s degree (*n =* 288, 85%). The three cross-sections of clinicians did not differ on age, gender, education, years of experience, or attitudes toward EBP (all *p*s > .25). The sample demographics broadly matches national demographics in mental health clinicians with regard to gender and ethnicity/race [73].

### Clinician participation in EBP initiatives

By study conclusion, 171 (50%) clinicians had participated in one or more EBP initiatives. Of this group, 100 clinicians (29%) had participated in one, 39 (12%) participated in two, 19 (6%) participated in three, ten (3%) participated in four, and three (1%) participated in five initiatives. See Table 1 for the average number of initiatives clinicians had participated in by wave.

### Trends in clinicians’ psychotherapy technique use over time

**Fig. 2:**
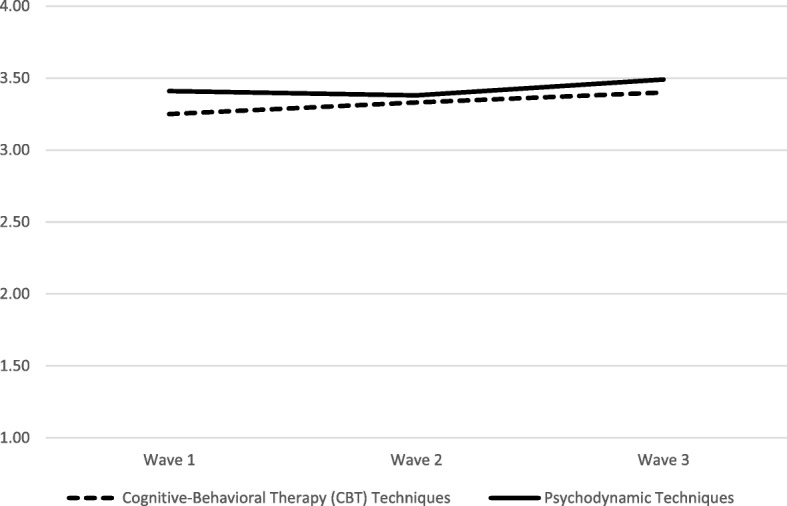
Unadjusted means of CBT and psychodynamic use at each wave

There was an increase in clinicians’ average use of CBT techniques across waves, controlling for covariates (*B*_*adj*_ = .09, *SE* = .04, *p* = .021); specifically, average CBT use increased by .09 points per wave, resulting in a 6% increase in clinicians’ average adjusted use of CBT techniques from wave 1 (*M* = 3.18) to wave 3 (*M* = 3.37). This represents a small standardized mean increase in CBT use of *d* = .29. In contrast, there was no observed change in clinicians’ average adjusted use of psychodynamic techniques (*B*_*adj*_ = .02, *SE* = .04, *p* = .570) from wave 1 (*M* = 3.37) to wave 3 (*M* = 3.41). Table 1 and Fig. 2 show the unadjusted means of CBT and psychodynamic use at each wave (Table 2).

**Table 2:** Effects of time and participation in EBP initiatives on clinicians’ use of evidence-based and non-evidence-based psychotherapy techniques

	Use of cognitive-behavioral techniques				Use of psychodynamic techniques			
	Model 1A		Model 2A		Model 1B		Model 2B	
	*B*	[95% CI]	*B*	[95% CI]	*B*	[95% CI]	*B*	[95% CI]
Time	.09*	[.02–.17]	.07	[− .01–.15]	.02	[− .06–.10]	.02	[− .06–.10]
Cumulative # of EBP initiatives			.09*	[.01–.16]			.01	[− .06–.09]

*K* = 20 organizations, *N* = 340 clinicians; *CBT* cognitive behavioral therapy, *EBP* evidence-based practice. All models control for organization size, clinician attitudes toward evidence-based practice, clinician participation in evidence-based practice initiatives upon study entry (yes/no), clinician education, years of experience, age, gender, and client age

*p < .05; **p < .01; ***p < .001

### Relation of EBP initiative participation to clinicians’ psychotherapy technique use

**Fig. 3:**
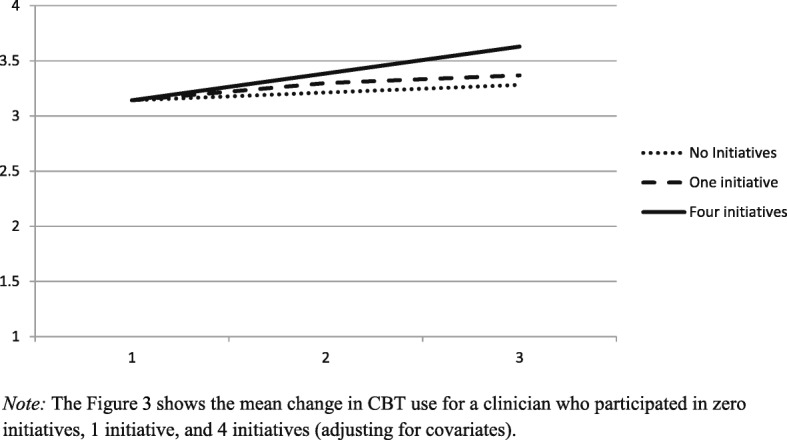
Adjusted mean change in CBT use for a clinician who participated in 0, 1, and 4 initiatives

Clinicians’ participation in EBP initiatives during the study was positively related to increased use of CBT techniques, controlling for wave and all covariates (*B*_*adj*_ = .09, *SE* = .04, *p* = .019). For each additional EBP initiative, clinician CBT technique use increased by 3%. For clinicians who did not participate in any EBP initiatives, average adjusted CBT use increased from wave 1 (*M* = 3.14) to wave 3 (*M* = 3.28) by *d* = .21, a small effect. On average, clinicians in the study participated in .85 EBP initiatives by the study’s end which corresponds to an adjusted average increase in CBT use from wave 1 (*M* = 3.14) to wave 3 (*M* = 3.36) of 7% or *d* = .32, a small effect. For clinicians who participated in two EBP initiatives by the study’s end, average adjusted CBT use increased from wave 1 (*M* = 3.14) to wave 3 (*M* = 3.46) by 10% or *d* = .47, a medium effect. For the 9.6% of clinicians who participated in three or more EBP initiatives by the study’s end, average adjusted CBT use increased by *d =* .61 (13%) or more, a medium-to-large effect. See Fig. 3. Participation in EBP initiatives was not related to clinicians’ psychodynamic technique use (*B*_*adj*_ = .01, *SE* = .04, *p* = .709).

### Associations among baseline organizational characteristics and trends in clinicians’ average use of psychotherapy techniques

Proficient organizational culture at baseline predicted variation in clinicians’ average CBT technique use over time (Table 3). Specifically, results from the mixed effects regression model indicated that organizations with more proficient cultures at baseline exhibited greater increases in clinicians’ average use of CBT techniques across waves (*B*_*adj*_ = .007, *SE* = .003, *p* = .048; percent change = 8%, *d* = .41) compared to organizations with less proficient cultures (percent change = − 2%, *d* = −.12). This effect remained significant after adjustment under the B-H false discover procedure. None of the organizational characteristics moderated the effect of time on clinicians’ average adjusted psychodynamic technique use either before or after the B-H correction (see Table 3).

**Table 3:** Relationships between baseline organizational characteristics and change in clinicians’ use of evidence-based and non-evidence-based psychotherapy techniques over 5 years

	Cognitive-behavioral technique use						Psychodynamic technique use					
					% Change from W1 to W3 (Cohen’s *d*)						% Change from W1 to W3 (Cohen’s *d*)	
Moderator	*B*	[95% CI]	*p*	*p*_*adj*_	Moderator = Low	Moderator = High	*B*	[95% *CI*]	*p*	*p*_*adj*_	Moderator = Low	Moderator = High
Proficient organizational culture					− 2% (*d* = − .12)	8% (*d* = .41)					− 3% (*d* = − .18)	4% (*d* = .20)
Main effect of moderator at W1	− .007	[− .023–.008]	.311				− .005	[− .018–.007]	.403			
Main effect of wave	.047	[− .041–.135]	.272				.003	[− .083–.090]	.936			
Moderator by wave interaction	.007^a^	[.001–.013]	.048	.144			.005	[− .002–.011]	.130	.334		
Implementation climate					3% (*d* = .14)	5% (*d* = .26)					3% (*d* = .17)	− 1% (*d* = −.05)
Main effect of moderator at W1	.090	[− .332–.511]	.660				.188	[− .138–.514]	.240			
Main effect of wave	.065	[− .022–.152]	.134				.020	[− .063–.103]	.618			
Moderator by wave interaction	.039	[− .133–.210]	.641	.641			− .072	[− .226–.081]	.334	.334		
Implementation Leadership					1% (*d* = .07)	8% (*d* = .38)					− 1% (*d* = −.08)	4% (*d* = .20)
Main effect of moderator at W1	− .064	[− .376–.247]	.669				− .145	[− .408–.117]	.258			
Main effect of wave	.072	[− .011–.155]	.086				.020	[− .062–.102]	.618			
Moderator by wave interaction	.073	[− .060–.207]	.263	.395			.065	[− .067–.198]	.312	.334		

*K* = 20 organizations, *N* = 340 clinicians. *CBT* cognitive behavioral therapy, *p*_*adj*_ *p* value adjusted using the Benjamini-Hochberg procedure with a false discovery rate of .25. *W1* wave 1; *W3* wave 3. Results based on three-level mixed-effects regression models with wave at level 1, clinician at level 2, and organization at level 3. All models control for organization size, clinician education, years of experience, age, gender, attitudes toward evidence-based practice, participation in evidence-based practice (EBP) initiatives upon study entry (yes/ no), and cumulative number of EBP initiatives participated in by wave, and client age. Low and high values of the moderator correspond to ± 1 SD, respectively

^a^Effect is statistically significant according to unadjusted *p* value and based on the Benjamini-Hochberg procedure

## Discussion

This study represents an opportunity to learn from a system encouraging EBP implementation [10, 14] and can inform future policy and research. First, in a public system supporting EBP implementation, EBP use increased over time and clinicians who participated in system-sponsored training initiatives increased their EBP use even more. Second, proficient organizational culture modified the effect of system efforts to increase implementation, which elucidates a potential future target for implementation strategy trials [28, 74]. While prior work has identified correlational associations between determinants like proficient organizational culture and outcomes, this study advances the field by prospectively elucidating the relationship between proficient culture and change over time. Despite our enthusiasm about these findings, it is important to note that they are preliminary given study limitations (i.e., self-reported measure of practice use and lack of a comparison system).

As expected, clinician use of EBPs modestly increased over the 5 years in which the system created a centralized infrastructure to de-silo EBP implementation [10]. Although only half the clinicians in the study participated in system-sponsored EBP initiatives, there was a significant increase in clinicians’ use of EBPs system-wide during the study period. Potential explanations include that supervisors trained in these EBPs through EBP initiatives may have supported clinicians not formally trained in applying these techniques, that peer interactions may have increased clinician interest in these techniques, or that the changing system culture might reflect new organizational priorities. Clinicians participating in system-sponsored EBP training initiatives increased their use of EBPs twice as much as those not formally trained. Although these increases are promising, the effects were not large in magnitude and raise questions about clinical significance. In a large system serving over 30,000 children and families annually, an increase of 6% might have a population mental health impact, but further research is needed to understand the clinical impact of small, system-wide increases in use of EBPs. Future studies evaluating the impact of system-wide implementation must include client outcomes using hybrid effectiveness-implementation designs [75] to ensure that the end goal of implementation efforts (i.e., client reach and outcomes) is achieved and that questions related to clinical significance and cost-effectiveness can be answered.

Consistent with the literature [53, 56, 76–78], clinicians working in organizations with more proficient cultures at baseline exhibited greater increases in CBT use. This extends previous findings by prospectively linking proficiency culture to increased EBP use over time. Clinicians working in such organizations may be more motivated to improve their competence in up-to-date practices and have more opportunities to participate in EBP training because of leaders’ EBP prioritization. Although proficient culture is a more distal construct on the causal implementation pathway, these preliminary results suggest the importance of attending to general organizational factors in the implementation process. Future work should clarify if proficient culture is a more powerful target for implementation efforts versus training and ongoing support initiatives or if they result in a synergistic effect.

Clinicians’ self-reported non-evidence-based technique use remained stable. Given that there is little knowledge of the effect of delivering EBPs alongside potentially contraindicated approaches [23], these findings point to the importance of attending to deimplementation [79, 80]. Further, this finding provides evidence of discriminant validity and suggests that the relationships observed between initiative participation and proficient organizational culture are not due to spurious findings or common method error variance [81].

Study methodological limitations include that this study was only conducted in one system and that CBT increases observed may be a national trend; that results may be influenced by cohort effects; that we relied on self-reported clinician use of techniques [82, 83] [84] rather than actual clinician behavior or patient outcomes; that the response rate was only 60%; that implementation strategies were not experimentally manipulated; and that these results may not generalize to smaller organizations and/or single clinician organizations given that we focused our sampling on organizations with larger programs. Study analytical limitations include that we conducted multiple tests of moderators given our exploratory aims, which increased the likelihood of a type I error; that the study may have been underpowered to detect effects of organizational moderators given the sample of 20 organizations at level 3; and that results included large confidence intervals on all effects that almost overlap with zero.

## Conclusions

Despite substantial efforts to implement EBPs over the past 20 years, downstream effects have rarely been systematically measured [85]. This study provides insight into clinician self-reported change in use of EBPs for youth with psychiatric disorders and suggests that system efforts to implement EBPs can result in modest clinician behavior change. It also suggests the potential importance of attending to organizational factors when targeting implementation strategies. It is commendable that systems are prioritizing EBPs and applying principles from implementation science to engender clinician behavior change. If the findings from this study are replicated in other settings, future research should develop implementation strategies that move beyond training and consultation to target and align system characteristics like policies and funding and organizational characteristics such as proficient cultures to be optimally effective [86].

## Abbreviations

CBT: Cognitive-behavioral therapy
DBHIDS: Philadelphia Department of Behavioral Health and Intellectual Disability Services
EBP: Evidence-based practice
EBPAS: Evidence-Based Practice Attitude Scale
EPIC: Philadelphia Evidence Based Practice and Innovation Center
ICS: Implementation Climate Scale
OSC: Organizational social context
TPC-FR: Therapy Procedures Checklist-Family Revised
