# Correction to: A repeated cross-sectional study of clinicians’ use of psychotherapy techniques during 5 years of a system-wide effort to implement evidence-based practices in Philadelphia

**DOI:** 10.1186/s13012-019-0936-9

**Published:** 2019-09-09

**Authors:** Rinad S. Beidas, Nathaniel J. Williams, Emily M. Becker-Haimes, Gregory A. Aarons, Frances K. Barg, Arthur C. Evans, Kamilah Jackson, David Jones, Trevor Hadley, Kimberly Hoagwood, Steven C. Marcus, Geoffrey Neimark, Ronnie M. Rubin, Sonja K. Schoenwald, Danielle R. Adams, Lucia M. Walsh, Kelly Zentgraf, David S. Mandell

**Affiliations:** 1 Department of Psychiatry, Perelman School of Medicine, University of Pennsylvania, Philadelphia, PA USA; 2 Department of Medical Ethics and Health Policy, Perelman School of Medicine, University of Pennsylvania, Philadelphia, PA USA; 3 Penn Implementation Science Center at the Leonard Davis Institute of Health Economics (PISCE@LDI), University of Pennsylvania, Hall- Mercer Community Mental Health Center, Philadelphia, PA USA; 4 School of Social Work, Boise State University, Boise, ID USA; 5 Department of Psychiatry, University of California San Diego, San Diego, CA USA; 6 Department of Family Medicine and Community Health, University of Pennsylvania Perelman School of Medicine, Philadelphia, PA USA; 7 American Psychological Association, Washington, DC USA; 8 Community Behavioral Health, Impact Reach, LLC, Philadelphia, PA USA; 9 Department of Behavioral Health, Philadelphia, PA USA; 10 Department of Child and Adolescent Psychiatry, New York University Langone Health, New York, NY USA; 11 School of Social Policy and Practice, University of Pennsylvania, Philadelphia, PA USA; 12 Oregon Social Learning Center, Eugene, OR USA; 13 School of Social Service Administration, University of Chicago, Chicago, IL USA; 14 Department of Psychology, University of Miami, Miami, FL USA; 15 Hall- Mercer Community Mental Health Center, 16Impact Reach, LLC, Philadelphia, PA USA

**Correction to: Beidas et al. Implement Sci (2019) 14:67**

**https://doi.org/10.1186/s13012-019-0912-4**

Following publication of the original article [1], the authors reported an error in the “Participants” sub-section. The original article states:

Over the course of the 5 years, we enrolled 21 out of the 29 organizations (73%; some organizations had more than one site, resulting in a total of 27 sites).

However, it should state:

Over the course of the 5 years, we enrolled **22** out of the 29 organizations (**76%**; some organizations had more than one site, resulting in a total of **31** sites).
